# Specificity of Pairing Afferent and Efferent Activity for Inducing Neural Plasticity with an Associative Brain–Computer Interface

**DOI:** 10.3390/s26020549

**Published:** 2026-01-14

**Authors:** Kirstine Schultz Dalgaard, Emma Rahbek Lavesen, Cecilie Sørenbye Sulkjær, Andrew James Thomas Stevenson, Mads Jochumsen

**Affiliations:** 1 Department of Health Science and Technology, Aalborg University, 9260 Gistrup, Denmark; 2 Center for Neurotechnology and Rehabilitation, Faculty of Medicine, Aalborg University, 9260 Gistrup, Denmark

**Keywords:** paired associative stimulation, neural plasticity, brain–computer interface, neurorehabilitation, movement-related cortical potentials, electrical stimulation

## Abstract

Brain–computer interface-based (BCI) training induces neural plasticity and promotes motor recovery in stroke patients by pairing movement intentions with congruent electrical stimulation of the affected limb, eliciting somatosensory afferent feedback. However, this training can potentially be refined further to enhance rehabilitation outcomes. It is not known how specific the afferent feedback needs to be with respect to the efferent activity from the brain. This study investigated how corticospinal excitability, a marker of neural plasticity, was modulated by four types of BCI-like interventions that varied in the specificity of afferent feedback relative to the efferent activity. Fifteen able-bodied participants performed four interventions: (1) wrist extensions paired with radial nerve peripheral electrical stimulation (PES) (matching feedback), (2) wrist extensions paired with ulnar nerve PES (non-matching feedback), (3) wrist extensions paired with sham radial nerve PES (no feedback), and (4) palmar grasps paired with radial nerve PES (partially matching feedback). Each intervention consisted of 100 pairings between visually cued movements and PES. The PES was triggered based on the peak of maximal negativity of the movement-related cortical potential associated with the visually cued movement. Before, immediately after, and 30 min after the intervention, transcranial magnetic stimulation-elicited motor-evoked potentials were recorded to assess corticospinal excitability. Only wrist extensions paired with radial nerve PES significantly increased the corticospinal excitability with 57 ± 49% and 65 ± 52% immediately and 30 min after the intervention, respectively, compared to the pre-intervention measurement. In conclusion, maximizing the induction of neural plasticity with an associative BCI requires that the afferent feedback be precisely matched to the efferent brain activity.

## 1. Introduction

Stroke remains one of the leading causes of acquired disability in adults [1], but despite ongoing rehabilitation efforts, many patients continue to suffer from impairments [2]. This is due to various degrees of impairment and difficulty in selecting the proper rehabilitation techniques that will be most efficient for recovering motor function of the individual patients [3,4]. Several rehabilitation techniques exist, but their efficacy varies [4]. Therefore, there is an incentive to develop new effective rehabilitation approaches. Effective rehabilitation should adhere to motor learning principles [5] and should promote neural plasticity, which is considered a key mechanism underlying motor learning [6].

Brain–computer interfaces (BCIs) have been proposed as a promising tool for promoting stroke recovery [7,8,9,10]. They can induce long-term potentiation-like plasticity by pairing activation in affected brain regions with congruent afferent feedback from the impaired limb, in a manner similar to paired associative stimulation [11,12]. This approach has consistently been shown to induce neural plasticity in both able-bodied participants and stroke patients [11,12,13,14] and has been associated with improvements in functional recovery [7,15].

The mechanism by which BCIs induce neural plasticity involves detecting movement-related brain activity and delivering precisely timed somatosensory feedback. When a user attempts a movement, the motor cortex is activated, producing a movement-related cortical potential (MRCP) and changes in the mu and beta rhythms known as event-related desynchronization and synchronization [16,17,18,19]. These signals can be recorded and extracted from electroencephalography (EEG) and used as control inputs for the BCI. Upon detection of an attempted movement, the BCI triggers feedback, such as electrical stimulation of muscles or nerves [7,14], or movement via an exoskeleton [20], or rehabilitation robot [9,21], to provide somatosensory feedback that matches the movement intention. This somatosensory feedback reaches the cortex in close temporal proximity to the efferent activity, a pairing proposed to induce long-term potentiation-like plasticity [22]. Notably, similar protocols have been implemented without real-time online BCI control [22] but using associative BCIs instead. These have also been shown to induce neural plasticity and promote functional recovery in stroke patients [14,23,24,25], with effects comparable to those achieved using online BCIs [14,26]. In the associative BCI paradigm, users perform 30–50 visually cued movements prior to training while EEG is recorded [22,23]. From this EEG data, the MRCP is extracted and the timing between its peak negativity and the visual cue is used to trigger electrical stimulation during the actual training. This approach assumes that the users will initiate the movements at the same time relative to the visual cue as they did in the calibration phase since the electrical stimulation is delivered independently of real-time intent. The effectiveness of associative BCI training is well-established, but there is still room for refinement. Importantly, the modality used to evoke somatosensory feedback, whether electrical stimulation or robotic assistance, does not appear to affect the degree of plasticity when paired with motor cortical activity [13]. However, pairing is essential; the relatively low number of peripheral stimuli alone (50–100) is insufficient to induce neural plastic changes without concurrent motor cortex activation [11,12,22].

Because the plastic changes induced by associative BCI protocols are thought to be long-term potentiation-like, the pairing between motor cortical activity and somatosensory feedback needs to be specific. Previous studies have tested this by pairing motor cortical activation associated with an agonist movement with electrical stimulation of the nerve innervating the antagonist muscle. These studies found no increases in corticospinal excitability for the target muscle [22]. However, the degree of specificity required for effective plasticity induction remains an open question, particularly in clinical populations where movement execution may be impaired or inconsistent.

Therefore, the current study examined how precisely efferent activity (i.e., movement intention) must align with somatosensory feedback to effectively induce plasticity. To ensure reliable execution of movements, able-bodied participants performed physical movements instead of motor imagery, which has commonly been employed in previous associative BCI protocols but poses challenges for experimental control. Accurately performing motor imagery of specific limb movements, such as wrist extension or palmar grasp, is challenging due to the lack of ground truth; users cannot verify whether the imagined movement is executed correctly. Furthermore, motor imagery is a skill that requires training and may vary across individuals. To address these limitations, this study aimed to test the specificity of the associative BCI protocol for inducing neural plasticity using executed rather than imagined movements. Executed movements were paired with somatosensory feedback via peripheral electrical nerve stimulation to assess changes in corticospinal excitability assessed by transcranial magnetic stimulation (TMS) under four conditions: (1) completely matching feedback, (2) non-matching feedback, (3) sham stimulation (no feedback), and (4) partially matching feedback.

## 2. Materials and Methods

### 2.1. Participants

Fifteen healthy participants were included in the study (eight women and seven men; mean age = 29 ± 9 years). Each participant provided written informed consent prior to participating in the study and filled out a questionnaire regarding eligibility for TMS based on the recommendation in [27]. The study was approved by the local ethical committee (N-20240040), and the study was conducted in accordance with the Declaration of Helsinki.

### 2.2. Experimental Setup

**Figure 1:**
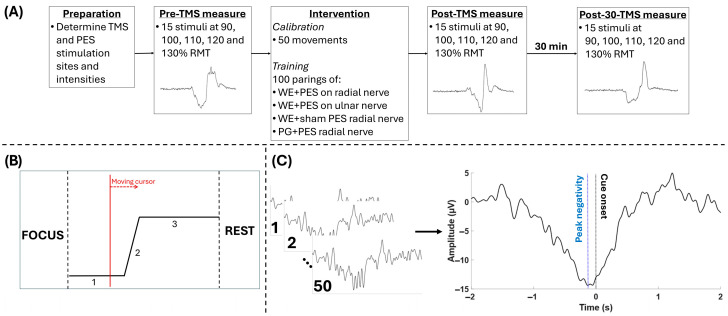
(**A**) Overview of the experiment. Initially, EMG electrodes were placed and the hotspot and resting motor threshold (RMT) for the transcranial magnetic stimulation (TMS) were determined. Also, the motor threshold (MT) or perception threshold (PT) for the peripheral electrical stimulation were determined depending on the intervention. The pre, post, and post-30 min measurements were identical. After the pre-intervention measurement, EEG was recorded while the participant performed 50 movements guided by the visual cue (**B**) to determine when to trigger the electrical stimulation in the following intervention. In the intervention, 100 movements (palmar grasps, PG, or wrist extension, WE) were paired with electrical stimulation. Four interventions were performed on four separate days. (**B**) Visual cue that was used for guiding the participants during the 50 movements and during the 100 pairings of movement and electrical stimulation. The preparation phase (1) lasted 1 s before the participant performed a ballistic low-force movement (2), which was maintained for 2 s (3). This was preceded by a 1 s warning (displayed on the screen) to focus the attention on the screen, and a 3 s rest period (displayed on the screen) followed the movement. (**C**) Extracting peak negativity from the calibration data to guide the electrical stimulation in the intervention. The cleaned EEG epochs were averaged, and the time between peak negativity (blue dashed line) and movement cue onset (marked with 2 in (**B**)) was measured. The time ‘0 s’ was when the movement cue was given.

The participants were seated comfortably in a chair and introduced to TMS. Each participant completed four different interventions using the right hand: (1) wrist extension (WE) paired with peripheral electrical stimulation (PES) on the radial nerve (matching feedback), (2) WE paired with PES on the ulnar nerve (non-matching feedback), (3) WE paired with sham PES on the radial nerve (no feedback), and (4) palmar grasp (PG) paired with PES on the radial nerve (partially marching feedback). The order of the interventions was randomized and were conducted on four different days, separated by at least 24 h. Each intervention consisted of 100 movements paired with PES (see Figure 1A). Prior the intervention, the participant performed 50 WEs (interventions 1–3) or PGs (intervention 4) while continuous EEG was recorded. The participants were visually cued to initiate the movement using a custom-made MATLAB script (The MathWorks, Natick, MA, USA). A moving cursor displayed when the participants should prepare to execute the movement (1 s), execute and movement and maintain the contraction (2 s). Before the moving cursor was displayed, “FOCUS” was displayed for one second for the participant to direct the attention to the forthcoming task, and after the movement was executed “REST” was displayed for four seconds (see Figure 1B). In the preparation phase the participants were instructed to prepare for executing the movement accurately with respect to the visual cue to initiate the movement. The movements were performed ballistically with a low force to reduce the risk of muscle fatigue. During the preparation and movement phases, the participants were instructed to avoid movements of the head and body and avoid blinking to reduce the effect of artifacts. During the resting phase, the participants were instructed to rest and blink and reposition themselves if needed. The 50 movements were then used to determine the mean peak negativity (PN) with respect to the visual cue for initiating the movement (see Figure 1C). This was used to deliver PES at the proper time with respect to the movement onset such that the afferent feedback from the electrical stimulation would reach the cortex when it was most active (at peak negativity). Thirty milliseconds were subtracted from the time of PN with respect to the visual cue to allow for the propagation time and processing delay in the brain. After determining the timing of the PES, the participants initiated the intervention. Prior to (pre), immediately after (post) and 30 min after (post-30) the intervention, motor-evoked potentials (MEPs) were elicited using TMS to assess changes in the corticospinal excitability. The MEPs were recorded from extensor carpi radialis longus (ECRL).

### 2.3. Stimulation

#### 2.3.1. Transcranial Magnetic Stimulation

MEPs were recorded using single-pulse TMS delivered with a figure-of-eight coil with a posterior–anterior current direction (Magstim, BiStim2, Dyfed, UK). A TMS tracking system was used to ensure the coil was placed at the same location throughout the experiment (POLARIS Optical Tracking System, NDI Northern Digital Inc., Waterloo, ON, Canada). First, the TMS hotspot was located where the largest peak-peak amplitudes of the MEPs were obtained in ECRL. Next, the resting motor threshold was determined. The resting motor threshold was determined using the open source TMS Motor Threshold Assessment Tool (MTAT 2.1 by Awiszus and Borckardt) to identify the lowest stimulation intensity needed to obtain peak-peak MEP amplitudes of 50 µV. 15 stimulations were recorded during the pre, post and post-30 TMS at five different intensities: 90%, 100%, 110%, 120%, and 130% of the resting motor threshold. Consecutive stimuli were separated by 5–7 s.

#### 2.3.2. Peripheral Electrical Stimulation

The PES was performed using a Digitimer Constant Current stimulator model DS7A (Digitimer Ltd., Welwyn Garden City, Hertfordshire, UK) and self-adhesive stimulation electrodes (SELF-ADHESIVE TENS/NMES/FES STIMULATING ELECTRODES, 3.2 cm, Dura-Stick Premium) placed over the radial or ulnar nerve. The proper stimulation site was determined by increasing the stimulation intensity from 0 mA in incremental steps of 1–2 mA until a visible wrist extension or wrist flexion (in the intervention where WE was paired with ulnar PES) was observed. Two different stimulation intensities were used in the study. During the interventions, the PES was set to 110% of motor threshold to ensure activation of the afferents carrying proprioceptive feedback, except for the intervention with WE paired with sham PES. In that intervention, the PES was set to 80% of perception threshold to ensure no activation of the sensory afferents carrying proprioceptive feedback. The motor threshold was found by palpating the tendon of the muscle that was innervated by the stimulated nerve. The stimulation intensity was decreased in 0.5–1 mA steps from the intensity associated with the visible activation of the wrist extension or wrist flexion. The perception threshold was defined as the lowest intensity where the participant could feel the stimulation. Once the threshold was determined, the stimulation intensity was turned up and down around the identified threshold to ensure it was correct.

### 2.4. Recordings

#### 2.4.1. EEG

Continuous EEG was recorded from Fp1, C3, C1, Cz, C2 and C4 (g.GAMMAcap and G.SCARABEO active electrodes, G.Tec, Graz, Austria), with respect to the International 10-20 System. The signals were recorded using a sample frequency of 512 Hz (g.HIAMP, G.Tec, Austria). The ground and reference electrodes were placed at AFz and the right earlobe, respectively. A digital trigger was sent to the EEG amplifier at the beginning of the preparation phase of the visual cue to divide the continuous EEG into epochs.

#### 2.4.2. EMG

MEPs were recorded using surface EMG electrodes (Neuroline 720, Ambu A/S, Ballerup, Denmark) placed on the right ECRL muscle. Two electrodes were positioned on the belly of the muscle in a bipolar derivation, identified by palpation, and a ground electrode was placed on the lateral condyle of the right humerus. The EMG signals were sampled at 3000 Hz, amplified with a gain of 2500 *v*/*v* and bandpass filtered from 16–470 Hz (BrainSight, Rogue Research Inc., Montreal, QC, Canada). The EMG was recorded in epochs ranging from 100 ms prior to 300 ms after the trigger to initiate the magnetic stimulus.

### 2.5. Data Analysis

#### 2.5.1. Determining Peak Negativity

To determine PN from the 50 calibration movements, the EEG from Cz was used. Initially, the continuous EEG was filtered between 0.1–10 Hz using a 4th order Butterworth bandpass filter in MATLAB version 2023b. The continuous EEG was divided into 4 s epochs, two seconds before and after the visual cue to initiate the movement. Epochs with amplitudes exceeding ±150 µV were removed, and the remaining epochs were averaged to identify the PN of the MRCP. The difference in time of PN and the visual cue onset to initiate the movement was used to determine the timing of the PES. As outlined above, the PES was timed 30 milliseconds prior to the time difference between the PN and the cue onset to initiate the movement.

#### 2.5.2. Motor-Evoked Potentials

The peak-to-peak amplitude of the MEPs was extracted for each stimulus at each stimulation intensity. The median of the 15 peak-to-peak amplitudes across each of the five stimulation intensities was used for further analysis. To establish a relationship between the magnetic stimulation intensities and the median MEP peak-to-peak amplitudes, the median peak-to-peak amplitudes were used as input for a Boltzman sigmoidal fit to establish a relationship between the magnetic stimulation intensities using the Levenberg-Marquard non-linear least-mean-squares algorithm [28]. Three parameters were extracted from the sigmoidal fit: maximum peak-to-peak MEP amplitude (MEPmax), intensity needed to obtain 50% of MEPmax (S50), and the slope. The parameters represent the maximal motor response (MEPmax) from the stimulation intensities, and the threshold (S50) and gain (slope) of the corticospinal neurons and the motoneuron pool.

### 2.6. Statistical Analysis

Initially, one-way repeated measures analysis of variance (ANOVA) tests were performed with intervention as the within-subjects factor (four levels: WE + PES at radial nerve, WE + PES at ulnar nerve, WE + sham PES at radial nerve, and PG + PES at radial nerve) for the following parameters: PN value, TMS resting motor threshold, pre-intervention MEPmax, pre-intervention S50, and pre-intervention slope. For the PES at the radial nerve at 110% motor threshold, the stimulation intensities were compared with a paired *t*-test. The motor threshold was not determined in the sham condition, and the perception threshold was not determined in the other conditions except the sham condition. Next, three two-way repeated measures ANOVA tests were performed for MEPmax, S50, and slope with intervention (four levels: WE + PES at radial nerve, WE + PES at ulnar nerve, WE + sham PES at radial nerve, and PG + PES at radial nerve) and time (three levels: Pre, post, and post-30) as factors. A significant interaction between intervention and time was followed up with a repeated measures ANOVA test for each intervention with time (3 levels: Pre, post, and post-30) as factor. When the assumption of normality was violated, the data were log10-transformed, and when the assumption of sphericity was violated the Greenhouse-Geisser correction was applied. Significance was assumed when *p* < 0.05. For the repeated measures ANOVA tests, significant test statistics were followed up with post hoc analyses using the Bonferroni correction to account for multiple comparisons.

## 3. Results

**Figure 2:**
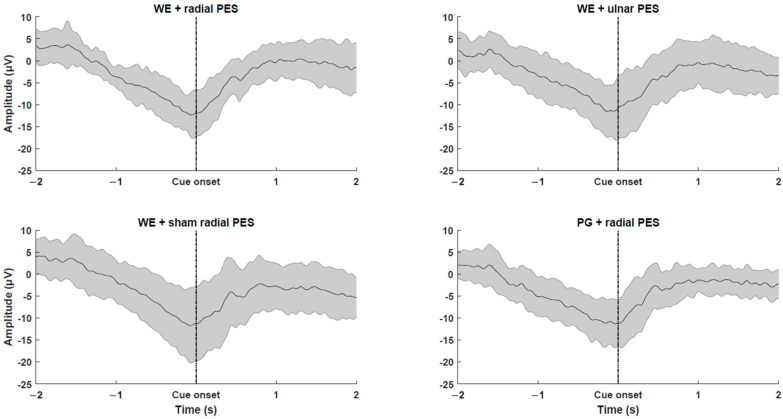
Grand average movement-related cortical potentials across all participants calculated from the calibration data collected prior the four interventions from the Cz channel. The shaded grey area represents the standard deviation across participants. Time ‘0 s’ is the visual cue to initiate the movement. WE: Wrist extension. PG: Palmar grasp. PES: Peripheral electrical stimulation.

The MRCPs across participants from the calibration data are presented in Figure 2. A clear increase in negativity can be observed when approaching the cue onset. Overall, the participants were accurate in timing their movement initiation to the visual cue, which was indicated by PN being close to the cue onset. The morphology of MRCP across the different interventions was similar. The mean PN time values across participants are presented in Table 1. The statistical analysis revealed that there was no difference in the PN time across the four interventions (F_(3,42)_ = 1.1; *p* = 0.37). Also, there was no significant difference in the resting motor threshold of the TMS (F_(1.9,25.1)_ = 1.7; *p* = 0.20), the motor threshold of the electrical stimulation (t_(14)_ = −0.56; *p* = 0.59), the pre-intervention measurements of MEPmax (F_(3,42)_ = 2.5; *p* = 0.07), the pre-intervention measurements of S50 (F_(3,39)_ = 1.5; *p* = 0.22), and the slope parameter of the Boltzman fit (F_(1.7,22.3)_ = 1.2; *p* = 0.32).

**Figure 3:**
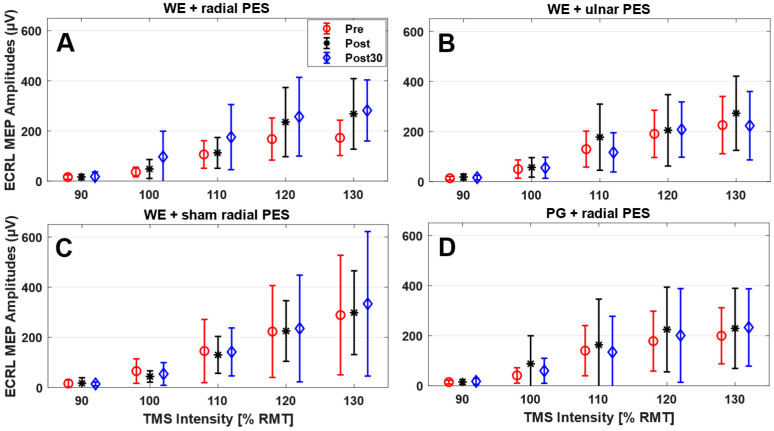
Overview of peak-to-peak motor-evoked potentials (MEPs) for the five transcranial magnetic stimulation (TMS) intensities. The values are presented as the mean value across participants ± standard deviation for the four interventions: Wrist extension + radial PES (**A**), wrist extension + ulnar PES (**B**), wrist extension + sham radial PES (**C**), and palmar grasp + radial PES (**D**). The black, blue, and red colour indicates, the pre-, post- and post-30 min-intervention measurements, respectively. WE: Wrist extension. PG: Palmar grasp. PES: Peripheral electrical stimulation. RMT: Resting motor threshold. ECRL: Extensor carpi radialis longus.

**Figure 4:**
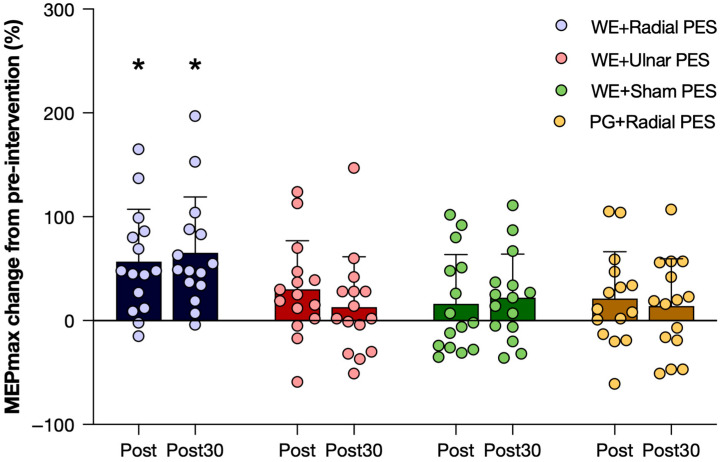
The relative change in the maximal motor-evoked potential (MEPmax) in percent from the transcranial magnetic stimulation measurement prior to the intervention to the measurement immediately after the intervention (Post) and 30 min after the intervention (Post30) for each of the four interventions. The results are presented as the mean value ± standard deviation across the participants, with each circle representing an individual participant. Note the significant (marked with ‘*’) increase from pre-intervention only for WE + Radial PES (matching feedback). WE: Wrist extension. PG: Palmar grasp. PES: Peripheral electrical stimulation.

The mean and standard deviation across participants of the peak-to-peak MEP amplitudes are presented in Figure 3, and the parameters extracted from the Boltzman fit are presented in Table 1. For all interventions, there was an increase in amplitude when the stimulation intensity increased, which was expected. It should be noted that there are considerable standard deviations, especially for the higher stimulation intensities showing that there is a high inter-participant variability. Greater post- and post-30 min-intervention measurements of the MEP amplitudes were obtained with respect to the pre-intervention measurements when WEs were paired with PES on the radial nerve (Figure 3A). This was also confirmed by the statistical analysis where there was a significant interaction between time and intervention (F_(6,84)_ = 2.8; *p* = 0.02) for MEPmax. Follow up tests revealed a significant effect of time when pairing WEs with PES of the radial nerve (F_(2,28)_ = 18.6; *p* < 0.001) where the post and post-30 measurements increased significantly with 57 ± 49% and 65 ± 52% from pre, respectively (see Figure 4). There was no effect of time when pairing WEs with PES on the ulnar nerve (F_(2,28)_ = 2.0; *p* = 0.16) or with sham PES on the radial nerve (F_(2,28)_ = 1.3; *p* = 0.29). Also, there was no effect when pairing PGs with PES on the radial nerve (F_(2,28)_ = 0.7; *p* = 0.53). For S50, there was no interaction between time and intervention (F_(6,78)_ = 1.1; *p* = 0.39), and no main effects of time (F_(2,26)_ = 2.2; *p* = 0.13) or intervention (F_(3,39)_ = 1.8; *p* = 0.17). Similarly for the slope parameter, there was no interaction between time and intervention (F_(3.7,47.6)_ = 0.9; *p* = 0.46) and no main effects of time (F_(1.4,17.7)_ = 0.6; *p* = 0.50) or intervention (F_(3,39)_ = 2.0; *p* = 0.13).

## 4. Discussion

This study demonstrated that only the pairing of WEs with radial nerve stimulation (matching feedback) led to an increase in peak-to-peak MEP amplitudes, highlighting the importance of precise somatosensory feedback in modulating corticospinal excitability with an associative BCI. Specifically, MEP amplitudes increased by 57% immediately after the intervention and with 65% after 30 min. These changes are consistent with previous studies using a similar associative BCI protocols, which have reported MEP increases ranging from 40–100% [11,12,13,22,26,29].

It is important to note that the variations in MEP amplitude changes across studies may result from methodological differences. These include the choice of target muscles (e.g., upper vs. lower limb), the type of efferent activity (motor execution vs. motor imagery), the number of pairings (typically 50–100), the feedback modality (e.g., electrical nerve stimulation, muscle stimulation, functional electrical stimulation, exoskeleton-/robot-assisted movements), and the specific stimulation parameters used to deliver afferent feedback. Additionally, differences in participant characteristics can contribute to variability. TMS-induced MEPs also exhibit substantial intra- and inter-participant variability, as reflected in the standard deviations in Figure 3 and Figure 4 and Table 1.

The interventions involving WEs paired with sham radial nerve stimulation (no feedback), antagonist (ulnar nerve) stimulation (non-matching feedback), or PGs paired with partially matching feedback resulted in modest non-significant increases in corticospinal excitability (10–30%). These finding align with previous studies that sham stimulation or antagonist nerve stimulation does not reliably enhance corticospinal excitability using associative BCI protocols [11,12,22]. This supports the notion that somatosensory feedback must be specific (i.e., homologous to the efferent activity) to effectively induce plasticity.

The lack of significant change in excitability following PGs paired with radial nerve stimulation may be explained by the simultaneous activation of both agonist and antagonist muscles. Prior research has shown that antagonist muscle activation can diminish the effect of electrical stimulation on the nerve supplying the agonist muscle [30]. Additionally, the small non-significant increments in corticospinal excitability observed across conditions may reflect a minor training effect from performing a total of 150 movements (50 during calibration and 100 during the intervention). Previous studies have reported mixed effect results regarding the impact of movements alone on corticospinal excitability, with effects likely depending on the number of repetitions. For example, 30 min of repetitive movement have been shown to increase corticospinal excitability by ~30% [30], whereas 50 repetitions did not increase the corticospinal excitability [29]. Finally, participants were required to time their movement onset to the visual cue, which demanded sustained attention. Attention has been shown to modulate corticospinal excitability, as reviewed in [31], and may have contributed to the observed variability.

The importance of specific somatosensory feedback in relation to efferent activity is consistent with the proposed mechanisms underlying associative BCI protocols, such as the one used in this study. The observed increases in corticospinal excitability exhibit characteristics similar to long-term potentiation, as previously suggested [22]. These include specificity, associativity, rapid onset, persistence after stimulation, and *N*-methyl-D-aspartate (NMDA) receptor involvement [32,33]. In this study, specificity was demonstrated by the fact that only matching feedback (WEs paired with radial nerve stimulation) significantly increased corticospinal excitability. Associativity was evident, as WEs paired with sham stimulation did not increase corticospinal excitability. The protocol also showed rapid onset, with effects emerging after a relatively short intervention (~15 min), and persistence, with excitability remaining elevated for at least 30 min post-intervention. Although NMDA-receptor involvement was not directly tested here, previous studies using paired-associative stimulation with TMS instead of movement to activate the motor cortex have shown that such plasticity is NMDA-dependent [34].

These findings, based on able-bodied participants, support the idea that training protocols should emphasise specificity. It should be noted that this was tested in a limited sample (15 participants) with younger able-bodied participants who are likely to respond differently to the training due to differences in age, motor capabilities and attention span, but there is also a potential for reaching a ceiling effect of the induction of plasticity with the able-bodied participants. The limited sample does not allow for an investigation of the effect of age and gender on the induction of neural plasticity. The findings in the current study should be validated in a stroke population although testing the research question, about the specificity of the somatosensory feedback with respect to the executed movement, would be challenging if it is not possible to control that the movements are performed correctly hence it would be a confounding factor. This will also be a challenge of providing specific feedback during the training in a stroke population is when the movement attempts are severely impaired and not accurately match the intended somatosensory feedback. While specific feedback may enhance rehabilitation outcomes, decoding intended movements from the brain activity may be necessary when execution is unreliable. Non-invasive BCIs have shown potential for decoding different movement types within the same limb (e.g., [35]), though with notable error rates, and functional movements have been decoded which will be more clinically relevant compared to single isolated movements for rehabilitating movements associated with activities of daily living [36]. An alternative approach could involve guiding patients to perform movements correctly using targeted cueing strategies and assume that the patients attempt to perform the correct movement. There is still an assumption that the users need to time their attempted movements consistently with respect to the cue and that they attempt to perform the intended movement that is being replicated for obtaining the proper specificity of the somatosensory feedback, but it is possible to avoid the technical issues of an online BCI, and they show comparable effects in terms of induction of neural plasticity [14,26].

## 5. Conclusions

Pairing WEs with matching feedback (radial nerve stimulation) significantly increased corticospinal excitability, whereas sham stimulation, antagonist (ulnar) stimulation, and partially matching stimulation paired with PGs did not produce significant changes. These findings highlight the importance of precise somatosensory feedback that accurately corresponds to the efferent brain activity to maximize plasticity. Future studies should validate this specificity requirement in individuals with motor impairments following stroke or other neurological conditions, such as multiple sclerosis, cerebral palsy, or incomplete spinal cord injury. These populations may benefit from associative BCI training, but the degree of feedback specificity required remains uncertain, especially when movement execution is impaired.

## Figures and Tables

**Table 1:** Overview of the peak negativity (PN) value [ms] with respect to the visual cue to initiate the movement (time: 0 ms), resting motor threshold (RMT) for the magnetic stimulation [% of maximum stimulator output, MSO], peripheral electrical stimulation (PES) intensity [mA], maximal peak-peak amplitude of the motor-evoke potential (MEPmax) [µV], transcranial magnetic stimulation intensity needed for obtaining 50% of MEPmax (S50) [% MSO], and slope parameter of the Boltzman fit for the four interventions. The values are presented as the mean value ± standard deviation across participants. WE: Wrist extension. PG: Palmar grasp. ’*’ marks a significant difference with respect to the pre-intervention measurement.

	WE + Radial PES	WE + Ulnar PES	WE + Sham Radial PES	PG + Radial PES
	Peak Negativity and stimulation parameters			
PN	−41 ± 99	−39 ± 149	21 ± 155	−52 ± 113
RMT	45 ± 10	47 ± 11	45 ± 10	46 ± 9
PES	16.3 ± 3.8	2.6 ± 0.8	0.7 ± 0.4	16.7 ± 3.3
	TMS results			
	Pre|Post|Post-30	Pre|Post|Post-30	Pre|Post|Post-30	Pre|Post|Post-30
MEPmax	191 ± 77|291 ± 139 *|309 ± 143 *	232 ± 112|287 ± 147|241 ± 136	296 ± 248|299 ± 174|342 ± 298	218 ± 124|262 ± 182|243 ± 170
S50	49 ± 12|50 ± 12|48 ± 12	51 ± 12|52 ± 12|52 ± 15	49 ± 12|49 ± 12|50 ± 12	49 ± 8|49 ± 12|50 ± 12
Slope	0.65 ± 0.58|0.64 ± 0.46|0.64 ± 0.46	0.42 ± 0.23|0.82 ± 0.89|0.64 ± 0.58	0.43 ± 0.31|0.41 ± 0.15|0.42 ± 0.23	0.53 ± 0.35|0.64 ± 0.62|0.51 ± 0.35

## Data Availability

The raw data supporting the conclusions of this article will be made available by the authors upon reasonable request.

## Abbreviations

The following abbreviations are used in this manuscript:

BCI	Brain–computer interface
PES	Peripheral electrical stimulation
MRCP	Movement-related cortical potential
EEG	Electroencephalography
TMS	Transcranial magnetic stimulation
WE	Wrist extension
PG	Palmar grasp
PN	Peak negativity
MEP	Motor-evoked potential
ECRL	Extensor carpi radialis longus
EMG	Electromyography
RMT	Resting motor threshold
MT	Motor threshold
PT	Perception threshold
MEPmax	Maximum peak-to-peak MEP amplitude
S50	Intensity needed to obtain 50% of MEPmax
ANOVA	Analysis of variance
